# 10-year cardiovascular disease risk and its predictors: a comparison between healthcare workers and the general public in Qatar

**DOI:** 10.1186/s12889-025-23999-0

**Published:** 2025-08-08

**Authors:** Sami Abdeen, Muna Abed Alah, Jihene Maatoug, Iheb Bougmiza, Nagah Selim

**Affiliations:** 1 Department of Preventative Health, Primary Health Care Corporation, Doha, Qatar; 2 Clinical Effectiveness Department, Primary Health Care Corporation, Doha, Qatar; 3 Preventive and Community Medicine Residency Program, Primary Health Care Corporation (PHCC), Doha, Qatar; 4 Faculty of Medicine of Sousse, University of Sousse, Sousse, Tunisia; 5 College of Medicine, QU Health, Qatar University, Doha, 2713 Qatar; 6 Department of Public Health and Preventive Medicine, Cairo University, Cairo, Egypt

**Keywords:** Atherosclerotic cardiovascular disease, 10-year risk, Lifestyle, General public, Health care workers, Qatar

## Abstract

**Background:**

The scientific literature presents conflicting findings on whether health care workers (HCWs) have better, or worse, cardiovascular health compared to the general population. The study aimed to compare the 10-year atherosclerotic cardiovascular disease (ASCVD) risk between the general public and HCWs in Qatar.

**Methods:**

An analytical cross-sectional study was conducted, involving two study groups: the general public and HCWs. Data collection included primary data collected through telephone interviews, as well as secondary data extracted from electronic health records. Combining these data, 10-year ASCVD risk was calculated using the American College of Cardiology/American Heart Association (ACC/AHA) risk calculator and compared between the two groups. Additionally, multivariable logistic regression analyses were performed to identify factors associated with higher risk among each group.

**Results:**

A total of 644 participants were included in this study, with 316 from the general public and 328 HCWs. The mean age of the participants was approximately 42 years, with females comprising 52.2% of the total sample. The general public had a significantly higher mean 10-year ASCVD risk than HCWs (8.0% vs. 3.4%, *p* < 0.001), even after adjusting for age and gender. Lifestyle emerged as the main independent predictor for higher ASCVD risk in both groups. Additionally, living alone and being a nurse were significant independent predictors of higher risk among HCWs.

**Conclusions:**

The study found that HCWs have significantly lower ASCVD risk than the general public, mainly due to their healthier lifestyle habits. These findings can help guide policymakers in developing national and community-based strategies to promote healthy lifestyles among the general public in Qatar.

## Introduction

Cardiovascular diseases (CVDs) are the leading cause of death globally (in low-, middle-, and high-income countries), taking an estimated 17.9 million lives each year [1]. CVDs have both modifiable and non-modifiable risk factors. Global comparative risk assessment studies have estimated that hundreds of thousands or millions of CVD deaths are attributable to established CVD risk factors (high blood pressure, high serum cholesterol, smoking, and high blood glucose), high body mass index, harmful alcohol use, some dietary and environmental exposures, and physical inactivity [2]. Assessment of ASCVD risk remains the foundation of primary prevention and is critical to identify patients who may benefit from more aggressive lifestyle interventions. Although all individuals should be encouraged to follow a heart-healthy lifestyle, estimating an individual’s ASCVD risk enables matching the intensity of preventive interventions to the patient’s absolute risk, to maximize anticipated benefit and minimize potential harm from overtreatment [3]. ASCVD risk estimation is used to guide decision making for many preventive interventions, including lipid management [4] and BP management [5]. A number of multivariate risk models have been developed for estimating the risk of initial ASCVD events in apparently healthy, asymptomatic individuals based upon assessment of multiple variables [6, 7]. One of the most used tools is the pooled cohort equations (PCE) which were introduced in 2013 for estimating short term (10-year) absolute rates of ASCVD events [8]. This tool is particularly important and most valid for individuals aged 40 to 79. As age is the primary determinant in these risk models, younger individuals often have lower calculated short-term (10-year) risk, which may lead to delays in initiating preventive strategies—potentially missing a critical window for effective intervention in those with traditional cardiovascular risk factors [9]. Therefore, for younger adults aged 20 to 39, assessing lifetime cardiovascular rather than short-term risk is recommended to guide early prevention efforts [10].

The scientific literature concerning the health care workers’ (HCWs’) health, specifically in relation to ASCVD, is marked by conflicting and inconsistent findings with several studies supporting the fact that HCWs do not take care of their own health any better than is to be expected due to their in-depth knowledge of health benefits and risks. In a national daily newspaper in November 2017, it was published that doctors die young compared to members of general public in Kerala [11]. While the life expectancy of an Indian adult is 67.9 years, and that of a “Malayali” (as a native of Kerala is called) is 74.9 years, mean ‘age of death’ of a Malayali doctor was 61.75 years. A study in Malaysia showed that 42% of HCWs had at least one medical condition, such as dyslipidemia (30.8%), hypertension (14.3%) or diabetes mellitus (10.4%) [12]. In another study by Mohd Ghazali et al., it was found that the majority (68.4%) of HCWs had at least three CVD risk factors with hypercholesterolemia and obesity being the most common [13]. On the other hand, other studies have suggested that HCWs have longer life expectancies and lower cardiovascular mortality rates compared with the general population. A study conducted in Canada found that HCWs had a lower prevalence of cardiovascular risk factors, and a reduced likelihood of major adverse cardiovascular events compared to the general population [14]. The findings indicated that HCWs had significantly lower rates of hypertension (16.9% vs. 29.6%), diabetes (5.0% vs. 11.3%), and smoking (13.1% vs. 21.6%), along with more favorable cholesterol profiles [14]. Furthermore, in a study by Erica Frank et al., it was found that the average age at death of US HCWs was older than for other same-race professionals and non-professionals [15].

Arabian Gulf region has seen major shifts in their demographic and lifestyle profile with reduced physical activity and increased consumption of calories and fat rich diets contributing to these trends [16, 17]. Consequently, hypertension, insulin resistance, diabetes, hyperlipidemia, smoking, and metabolic syndrome, have all increased [17, 18]. As a result, these countries are challenged by a major and increasing burden of CVDs. In Qatar, a study showed that approximately 16% of the total study population had one or more non-communicable disease (NCD) [19]. Type 2 Diabetes (T2D) and CVD are the greatest contemporary health challenges for Qatar [20]. To our knowledge and after extensive literature review, we found no studies done in the region and particularly in Qatar to assess ASCVD risk and compare it between HCWs and the general population. In our study we aimed to identify the disparities in ASCVD risk and its associated factors between the HCWs and the general population.

## Materials and methods

### Study design, setting, and the target population

This analytical cross-sectional study was conducted at Hamad Medical Corporation (HMC) which is the main public sector provider of secondary and tertiary healthcare services in the State of Qatar. The study involved two groups: the general public and healthcare workers (HCWs). Participants from each group were selected using simple random sampling using a computer-generated random number algorithm with the assistance of the Business Intelligence Unit (BIU). General public participants were randomly selected from individuals with a Health Card (HC) number in HMC database. Since all individuals residing in Qatar, including both nationals and residents, are assigned a HC number, this approach effectively represents the general population. HCWs participants were randomly selected from a list of all staff members currently employed at HMC.

The study included individuals aged 40–75 with available total cholesterol, High-Density Lipoprotein (HDL), and Low-Density Lipoprotein (LDL) lab results at HMC completed during the year prior to the start of data collection. Exclusion criteria included participants with a history of CVD, those with LDL levels over 190 mg/dL (as the used short-term 10-year ASCVD risk is not valid for LDL more than 190 mg/dL [21, 22]), and individuals in the general public group who are employed in healthcare professions.

### Sample size calculations

To achieve 80% power at a 5% significance level (two-sided) for detecting a 1-unit difference (equivalent to a 1% change in 10-year ASCVD risk), the required sample size was calculated to be 277 for each group, using the following formula [23–25]: *n = (Zα/2 + Zβ)² × 2σ²/d²*.

where *Zα/2* = 1.96, *Zβ* = 0.84, *σ²* is the population variance, and *d* is the detectable difference.

Additionally, to detect a 0.08 difference in the proportions of intermediate-to-high 10-year ASCVD risk between the two groups (general public and HCWs), with 80% power and a 5% two-sided significance level, the required sample size was calculated to be 316 for each group, using the following formula [26–28]: *n = (Zα/2 + Zβ)² × [p₁(1–p₁) + p₂(1–p₂)]/(p₁–p₂)²*.

where *p₁* and *p₂* represent the expected proportions in the two groups.

### Data collection and study variables

Data collection took place from October 2023 to January 2024 and involved secondary data extracted from the electronic health records system (Cerner) with support from the Business Intelligence Unit (BIU) at HMC. This data included measurements, and lab results necessary to calculate the 10-year ASCVD risk using the ACC-AHA calculators, such as weight and height, systolic and diastolic blood pressure, and lipid profile (total cholesterol, HDL, and LDL) from within the past year. In addition, primary data was gathered through telephone interviews with the selected participants using a structured questionnaire.

The questionnaire consisted of 3 sections:

* Section A, titled as “Sociodemographic and work-related information” that included general items (age, gender, nationality, education, marital status, living arrangements and sufficiency of family income). In addition, in this section we assessed the participants’ social support using Oslo Social Support Scale (OSSS-3). OSSS-**3** is a valid and reliable economic tool developed by Kocalevent in 2018 to evaluate the level of social support [29]. The OSSS-3 comprises of three items that assess the number of close confidents, the sense of concern from other people, and the relationship with neighbors, focusing on the availability of practical help. The sum score ranges from 3 to 14, with low values indicating poor levels and high values indicating strong levels of social support. The OSSS-3 sum score can be operationalized into three broad categories of social support: 3–8, poor social support; 9–11, moderate social support; and 12–14, strong social support.
* Section B, titled as “Health related information” that included questions about past medical history, medications history and family history of premature CVD.
* Section C, titled as “Lifestyle related information”. This section included items that were adapted from multiple valid and reliable tools as follows:
  I. The Simple Lifestyle Indicator Questionnaire (SLIQ) which is validated, reliable and sensitive-to-change tool [30–32] used to assess multiple lifestyle components. It has 5 components: diet (3 questions), physical activity (3 questions), alcohol consumption (3 questions), smoking (2 questions), and stress (1 question). For each component, a raw score and a category score can be calculated. For example, the diet raw score is the sum of three questions, concerning consumption of vegetables, fruits, and grains and their uptake frequency, which are each scored from 0 to 5. Then, raw scores can be categorized from zero to two (0 = score 0 to 5, indicating poor dietary habits, 1 = score 6 to 10, indicating an intermediate dietary habit, and 2 = score 11 to 15, indicating healthy dietary habits). This categorized score is known as the “diet category score”. Questions related to physical activity explore the type, intensity (light, moderate and vigorous), and frequency of physical activities practiced by the individual. Physical activity-related scores can be categorized into 0 (unhealthy lifestyle for physical activity), 1 (intermediate lifestyle for physical activity) and 2 (healthy lifestyle for physical activity). This categorized score is known as the “physical activity category score”. Alcohol ingestion-related questions explored alcohol consumption in terms of alcoholic drinks ingested per week. The raw score can be converted into the “alcohol category score” applying the following formula: in the case of 14 or more drinks per week, the score is coded as zero and indicates unhealthy drinking habits; in the case of 8–13 weekly drinks, the score is categorized as 1 and indicates an intermediately healthy drinking habits; and finally, in the case of 0–7 drinks per week, the category score is 2, indicating healthy drinking habits. Smoking related questions investigated current and former smoking habits. The score is categorized as 0 if the participant is a current smoker, 1 if the individual is a past smoker, and 2 if the person has never smoked. Finally, life stress is measured on a Likert scale ranging from 1 (“not at all stressful”) to 6 (“very stressful”). Scores from 1 to 2, from 3 to 4, and from 5 to 6 indicate unhealthy, intermediate, and healthy stress lifestyles, respectively. To provide equal weighting for each component, the overall SLIQ score is based on the 5 category scores. Each component has a category score of 0, 1, or 2, so overall SLIQ scores can range from 0 to 10. The higher the score, the healthier the lifestyle. A person is considered “unhealthy” if they have a SLIQ score of between 0 and 4, “intermediate” if the SLIQ score is between 5 and 7, and “healthy” if they score between 8 and 10 on the SLIQ [30].
  II. Perceived Stress Scale (PSS-4) that is a short, reliable, and valid tool to assess stress [33–35]. The PSS-4 consists of four questions that assess the participant’s feelings and thoughts over the past month on a 5-point Likert scale (Never, Almost never, Sometimes, Fairly often, Very Often). Questions 2 and 3 are reverse coded. The Total score is determined by adding together the scores of each of the four items the higher the score the more stress the participant has.
  III. Sleep duration in hours.

### Statistical analysis

The data was analyzed using IBM SPSS Statistics for Windows, version 26.0 (IBM, Armonk, NY). Frequencies and percentages were used to describe categorical data, while numerical data was described using the mean and standard deviation. The Mann-Whitney test was used to compare the SLIQ score and 10-year ASCVD risk between HCWs and the general public groups, as well as to compare proportions of binary outcomes across different levels of ordinal independent variables. Additionally, Ranked Analysis of Covariance (ANCOVA), also known as Quade’s test, was used to compare the 10-year ASCVD risk between HCWs and the general public while adjusting for age. The Chi-square and Fisher exact tests were employed to determine the differences in the proportions of different 10-year ASCVD risk groups across different levels of nominal independent variables as appropriate. Univariable and multivariable logistic regression analyses were used to assess the associated factors and predictors of higher 10-year ASCVD risk for both groups. The associations between predictors and outcomes were presented using adjusted odds ratios (AORs) and 95% confidence intervals (95% CIs). Statistical significance was considered at *p* < 0.05.

## Results

### Sociodemographic characteristics and background information

A total of 644 completed questionnaires were collected during the telephone interviews. Out of the total participants, 316 were from the general public, and 328 were currently employed as HCWs at HMC. As shown in Table 1, the mean age of the participants in the general public group was 52, while in the HCWs was 48. In terms of gender distribution, the HCWs group had a higher proportion of females, accounting for 62.2%, compared to the general public group, 39.6%. Participants represented 53 different nationalities, with Indian, Filipino, Qatari, and Egyptian nationalities being the most common. All participants in the HCWs group held a college or higher educational degree, whereas approximately two thirds of the general public group had attained this level of education. In both groups, over 90% of the participants were married, and more than three-quarters were living with their families. Among the HCWs group, 49.4% were from nursing and midwifery, 15.5% were physicians, and 35.1% were allied health professionals. Additionally, 57.7% of all participants had at least one chronic disease, with diabetes and hypertension being the most prevalent, affecting 34.9% and 29.0% of participants, respectively. Furthermore, 75.6% of participants were found to be overweight or obese, Supplementary Table S1.

**Table 1:** Sociodemographic characteristics of the general public group, HCWs group and the total sample

Characteristic		General public group (*n* = 316) No (%)	Healthcare workers group (*n* = 328) No (%)	Total sample (*N* = 644) No (%)
Age (in years)		52 ± 9	48 ± 6	50 ± 8
Gender	Female	125 (39.6)	204 (62.2)	329 (51.1)
	Male	191 (60.4)	124 (37.8)	315 (48.9)
Nationality	Indian	64 (20.3)	145 (44.2)	209 (32.5)
	Filipino	25 (7.9)	69 (21)	94 (14.6)
	Qatari	67 (21.2)	9 (2.7)	76 (11.8)
	Egyptian	34 (10.8)	21 (6.4)	55 (8.5)
	Others	126 (39.9)	84 (25.6)	210 (32.6)
Education	No formal education	9 (2.8)	0 (0)	9 (1.4)
	Primary/preparatory school	29 (9.2)	0 (0)	29 (4.5)
	Secondary/high school	69 (21.8)	0 (0)	69 (10.7)
	College or higher	209 (66.1)	328 (100)	537 (83.4)
Marital status	Not married	21 (6.6)	14 (4.3)	35 (5.4)
	Married	295 (93.4)	314 (95.7)	609 (94.6)
Living arrangement	Living alone	27 (8.5)	27 (8.2)	54 (8.4)
	Living with family	246 (77.8)	297 (90.5)	543 (84.3)
	Living with friends or roommates	43 (13.6)	4 (1.2)	47 (7.3)
Perceived sufficiency of monthly income	Not enough at all	22 (7)	25 (7.6)	47 (7.3)
	Just barely enough	53 (16.8)	68 (20.7)	121 (18.8)
	Enough	235 (74.4)	231 (70.4)	466 (72.4)
	More than enough	6 (1.9)	4 (1.2)	10 (1.6)
Social support (OSSS-3)	Poor	44 (13.9)	47 (14.3)	91 (14.1)
	Moderate	222 (70.3)	209 (63.7)	431 (66.9)
	Strong	50 (15.8)	72 (22)	122 (18.9)

### Lifestyle comparison between the two groups

As shown in Table 2, HCWs demonstrated significantly healthier dietary habits, with a greater proportion adhering to a healthy diet (18.3% vs. 14.2%). HCWs exhibited healthier smoking behaviors, with fewer smokers (4.3% vs. 13.9%) and a larger percentage of participants classified under healthy smoking category (88.7% vs. 79.1%, *p* < 0.001). Stress levels showed no significant differences between the groups. Overall, HCWs had a higher proportion of participants with a healthy lifestyle (15.9% vs. 10.4%, *p* < 0.001) with a significantly higher mean total SLIQ score (6.3 vs. 5.9, *p* = 0.002), though the general public reported a significantly longer average sleep duration (6.80 vs. 6.45 h, *p* < 0.001).

**Table 2:** Comparison of the lifestyle components between the general public and HCWs groups

Characteristic				General public group (*n*=316) No (%)	Healthcare workers group (*n*=328) No (%)	*P*-value*	Total No (%)
Simple Lifestyle Indicator Questionnaire (SLIQ)	Diet	Category score	Unhealthy	81 (25.6)	46 (14)	0.001	127 (19.7)
			Intermediate	190 (60.1)	222 (67.7)		412 (64)
			Healthy	45 (14.2)	60 (18.3)		105 (16.3)
		Row score M±SD		7.25±2.82	8.11±2.79	<0.001	7.69±2.83
	Physical activity	Category score	Unhealthy	263 (83.2)	256 (78)	0.087	519 (80.6)
			Intermediate	41 (13)	52 (15.9)		93 (14.4)
			Healthy	12 (3.8)	20 (6.1)		32 (5)
		Row score M±SD		2.57±3.65	3.05±3.65	0.017	2.81±3.66
	Alcohol	Category score	Unhealthy	0 (0)	0 (0)	0.326	0 (0)
			Intermediate	0 (0)	1 (0.3)		1 (0.2)
			Healthy	316 (100)	327 (99.7)		643 (99.8)
		Row score M±SD		0.15±0.86	0.14±0.92	0.910	0.14±0.89
	Smoking	Category score	Unhealthy	44 (13.9)	14 (4.3)	<0.001	58 (9)
			Intermediate	22 (7)	23 (7)		45 (7)
			Healthy	250 (79.1)	291 (88.7)		541 (84)
		Row score M±SD		1.65±0.71	1.84±0.47	<0.001	1.75±0.61
	Stress	Category score	Unhealthy	52 (16.5)	64 (19.5)	0.274	116 (18)
			Intermediate	148 (46.8)	154 (47)		302 (46.9)
			Healthy	116 (36.7)	110 (33.5)		226 (35.1)
		Row score M±SD		4±1	4±1	0.243	4±1
	Total	Total SLIQ category	Unhealthy	48 (15.2)	27 (8.2)	0.002	75 (11.6)
			Intermediate	235 (74.4)	249 (75.9)		484 (75.2)
			Healthy	33 (10.4)	52 (15.9)		85 (13.2)
		Total SLIQ score M±SD		5.95±1.37	6.3±1.28	0.002	6.13±1.33
PSS-4 score M±SD				3.49±2.56	3.34±2.38	0.591	3.41±2.47
Sleep duration (in hours) M±SD				6.8±2	6.5±1	0.002	7±1

*Abbreviations*: *SLIQ* Simple lifestyle indicator questionnaire, *PSS* Perceived Stress Scale

*Using Mann-Whitney test

### 10-year ASCVD risk

**Fig. 1:**
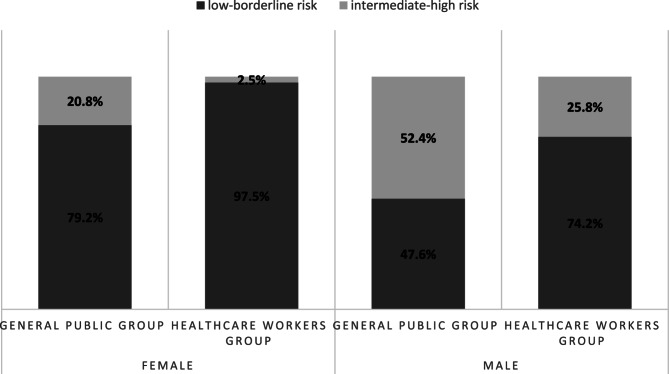
10-year ASCVD risk among the general public and HCWs groups stratified by gender

The general public group exhibits a 10-year ASCVD risk of more than double that of the HCWs group (8.0% (95% CI 7.0–9.0) vs. 3.4% (95% CI 2.9–3.9), respectively; *p* < 0.001). After stratifying by gender to account for different gender distributions between the groups, we found that among males, the general public group had a mean risk of 10.2% (95% CI 8.9–11.5), while the HCWs group had 5.9% (95% CI 4.9–6.9) with *p* < 0.001. Among females, the general public group had a mean risk of 4.6% (95% CI 3.4–5.8%), while the HCWs group had 1.8% (95% CI 1.6–2.1%) with *p* < 0. 001.Even after adjusting for age as a covariate the differences persisted. Among males, the risk was 9.0% (95% CI 8.1–9.9) in the general public group compared to 7.6% (95% CI 6.4–8.6) in the HCWs group (*p* = 0.041). Among females, the risk was 3.4% (95% CI 2.8–3.9) in the general public group and 2.6% (95% CI 2.2–3.1%) in the HCWs group (*p* = 0.049). In Fig. 1, the proportion of individuals with intermediate to high 10-year ASCVD risk was lower among males in the HCWs group (25.8%) compared to the general public group (52.4%). Similarly, among females, only 2.5% of the HCWs group were classified as having intermediate to high risk, compared to 20.8% in the general public group.

### Factors associated with intermediate to high 10-year ASCVD risk among general public groups

Among the general public group, the multiple logistic regression analysis, adjusting for non-modifiable risk factors such (age, gender, and race), revealed that only lifestyle was an independent predictor of the 10-year ASCVD risk. Participants with an unhealthy lifestyle were 3.3 times more likely to have an intermediate-high risk compared to those with an intermediate lifestyle (AOR 3.3, 95% CI: 1.30–9.09, *p* = 0.013), and 5.6 times more likely compared to those with a healthy lifestyle (AOR 5.56, 95% CI: 1.26-25.00, *p* = 0.023) (Table 3). The logistic regression model demonstrated a good fit to the data (Omnibus test: χ² = 199.07, *p* < 0.001; Hosmer–Lemeshow test: *p* = 0.672), with Nagelkerke R² of 0.632.

**Table 3:** The associated factors and predictors of intermediate to high 10-year ASCVD risk among the general public group using simple (univariable) and multivariable logistic regression analyses

Characteristics		Intermediate to high 10-year ASCVD risk				
		Univariable logistic regression			Multivariable logistic regression	
		Unadjusted OR (95%CI)	*P*-value	F test *P*-value*	AOR (95%CI)	*P*-value
Nationality	Indian	1.74 (0.95-3.2)	0.075	0.460	-------	-----
	Filipino	1.16 (0.48-2.79)	0.741		-------	-----
	Qatari	1.1 (0.6-2.03)	0.753		-------	-----
	Egyptian	0.95 (0.43-2.09)	0.896		-------	-----
	Other countries	1 [Reference]			-------	-----
Marital status	Not married	1.14 (0.47-2.79)		0.773	-------	-----
	Married	1 [Reference]	0.773		-------	-----
Living arrangement	Living alone	1 [Reference]		0.211	1 [Reference]	
	Living with family	0.75 (0.33-1.66)	0.475		0.78 (0.27-2.27)	0.645
	Living with friends or roommates	1.31 (0.5-3.44)	0.584		1.4 (0.38-5.18)	0.610
Perceived sufficiency of monthly income	Not enough at all	1 [Reference]		0.964	-------	-----
	Just barely enough	1.03 (0.37-2.82)	0.962		-------	-----
	Enough	0.93 (0.38-2.26)	0.872		-------	-----
	More than enough	1.44 (0.24-8.84)	0.691		-------	-----
Education level	No formal education	3.29 (0.8-13.53)	0.099	0.115	1.97 (0.3-12.93)	0.482
	Primary/preparatory school level	2.03 (0.93-4.43)	0.077		1.03 (0.34-3.15)	0.960
	Secondary/high school level	0.93 (0.53-1.64)	0.816		0.97 (0.4-2.34)	0.937
	College or higher	1 [Reference]			1 [Reference]	
Employment	Not Employed	0.91 (0.56-1.49)	0.715	0.715	-------	-----
	Employed not medical	1 [Reference]			-------	-----
Social support	Poor	1.48 (0.64-3.4)	0.362	0.620	-------	-----
	Moderate	1.32 (0.7-2.52)	0.393		-------	-----
	Strong	1 [Reference]			-------	-----
BMI		0.97 (0.94-1.01)	0.142	0.142	1.01 (0.95-1.07)	0.742
Lifestyle	Unhealthy lifestyle	1 [Reference]		0.001	1 [Reference]	
	Intermediate lifestyle	0.41 (0.22-0.78)	0.007		0.3 (0.11-0.77)	0.013
	Healthy lifestyle	0.15 (0.05-0.42)	<0.001		0.18 (0.04-0.79)	0.023
PSS score		1.01 (0.93-1.11)	0.781	0.781	-------	-----
Sleep duration		0.87 (0.75-1.01)	0.065	0.065	0.98 (0.8-1.2)	0.829

*Abbreviations*: *AOR* Adjusted odds ratio, *CI* Confidence interval, *BMI* Body mass index, *SLIQ* Simple lifestyle indicator questionnaire, *PSS* Perceived stress scale

*Variables with *P*-values ≤0.25 in the univariable analysis were included in the multivariable analysis

### Factors associated with intermediate to high 10-year ASCVD risk among HCWs group

Among the HCWs group, the multiple logistic regression analysis, adjusting for non-modifiable risk factors, showed that the living arrangement, medical profession, and lifestyle status were independent predictors of the 10-year ASCVD risk. Participants who live alone were 7.1 times more likely to have intermediate-high risk as compared to those who live with their families (AOR 7.14, 95% CI: 1.15-50.00, *p* = 0.034). In addition, multiple regression showed that nursing and midwifery medical professions were 11.1 times and 5.5 times more likely to have an intermediate-high risk compared to physicians and allied health professionals respectively ([AOR 11.11, 95% CI: 1.75–100.00, *p* = 0.011], [AOR 5.55, 95% CI: 1.11-25.00, *p* = 0.037]). This was despite univariable analysis showing that physicians had a higher proportion and odds of intermediate-to-high ASCVD risk. This discrepancy is mainly attributed to the significantly higher proportion of males (75% vs. 14%) and the higher mean age (50 years Vs 47 years) among physicians compared to nursing and midwifery medical professions both of which increase the calculated risk. Additionally, participants with unhealthy lifestyle were 10.0 times more likely to have an intermediate-high risk compared to those with an intermediate lifestyle (AOR 10.00, 95% CI: 1.96–100, *p* = 0.006), and 50.0 times more likely compared to those with healthy lifestyle (AOR 50.00, 95% CI: 4.00-500, *p* = 0.002) (Table 4). The logistic regression model demonstrated a good fit to the data (Omnibus test: χ² = 150.56, *p* < 0.001; Hosmer–Lemeshow test: *p* > 0.999), with Nagelkerke *R*² of 0.734.

**Table 4:** The associated factors and predictors of intermediate to high 10-year ASCVD risk among the HCWs group using simple (univariable) and multivariable logistic regression analyses

Characteristics		Intermediate to high 10-year ASCVD risk				
		Unadjusted OR (95%CI)	*P*-value	F test *P*-value*	AOR (95%CI)	*P*-value
Nationality	Indian	0.23 (0.1-0.53)	0.001	0.005	1.96 (0.7-10.46)	0.430
	Filipino	0.27 (0.09-0.76)	0.013		0.67 (0.09-4.94)	0.693
	Qatari	0.43 (0.05-3.64)	0.437		0.39 (0-566620.04)	0.897
	Egyptian	0.8 (0.24-2.68)	0.724		0.38 (0.32-4.66)	0.452
	Other countries	1 [Reference]			1 [Reference]	
Marital status	Not married	1.29 (0.28-5.98)		0.748	-------	-----
	Married	1 [Reference]	0.748		-------	-----
Living arrangement	Living alone	1 [Reference]		0.007	1 [Reference]	
	Living with family	0.31 (0.12-0.79)	0.015		0.14 (0.02-0.87)	0.034
	Living with friends or roommates	2.86 (0.34-24.3)	0.336		1.72 (0-228202.24)	0.928
Perceived sufficiency of monthly income	Not enough at all	1 [Reference]		0.694	-------	-----
	Just barely enough	3.66 (0.44-30.49)	0.230		-------	-----
	Enough	3.31 (0.43-25.43)	0.250		-------	-----
	More than enough	0 (0-0)	0.999		-------	-----
Medical profession	Nursing and midwifery	1 [Reference]		0.001	1 [Reference]	
	Physicians	4.00 (1.76-9.12)	0.001		0.09 (0.01-0.57)	0.011
	Allied health professionals	1.01 (0.43-2.35)	0.988		0.18 (0.04-0.90)	0.037
Social support	Poor	0.41 (0.08-2.08)	0.283	0.179	0.29 (0.02-3.42)	0.324
	Moderate	1.5 (0.63-3.58)	0.366		1.57 (0.27-9.00)	0.613
	Strong	1 [Reference]			1 [Reference]	
BMI		1.03 (0.97-1.10)	0.454	0.454	-------	-----
Lifestyle	Unhealthy lifestyle	1 [Reference]		0.031	1 [Reference]	
	Intermediate lifestyle	0.29 (0.11-0.76)	0.012		0.10 (0.02-0.51)	0.006
	Healthy lifestyle	0.52 (0.17-1.63)	0.262		0.02 (0.002-0.25)	0.002
PSS score		0.85 (0.71-1.01)	0.052	0.052	0.96 (0.72-1.27)	0.747
Sleep duration		1.2 (0.89-1.63)	0.234	0.234	1.66 (0.87-3.19)	0.127

*Abbreviations*: *AOR* Adjusted odds ratio, *CI* Confidence interval, *BMI* Body mass index, *SLIQ* Simple lifestyle indicator questionnaire, *PSS* Perceived stress scale

*Variables with *P*-values ≤0.25 in the univariable analysis were included in the multivariable analysis

## Discussion

Assessment of ASCVD risk remains the foundation of primary prevention and is critical to identify patients who may benefit from more aggressive lifestyle interventions. While several studies have examined cardiovascular risk factors in HCWs, there remains limited research on whether HCWs have a distinct cardiovascular risk compared to the general public. This study aims to compare the 10-year ASCVD risk and its associated factors among the general public and HCWs in Qatar.

The study investigated lifestyle components among HCWs and the general public. In our study, HCWs demonstrated an overall healthier lifestyle compared to the general public, primarily due to better diet and healthier smoking habits. This finding is consistent with studies from the USA, and Canada [36–39]. This can be explained by several factors inherent to the healthcare profession. Firstly, HCWs typically have greater knowledge and awareness of the importance of maintaining a healthy lifestyle due to their medical training and professional environment [40]. Additionally, the healthcare setting often promotes a culture of health, with more access to resources such as wellness programs, fitness facilities, and health-related information [40]. Moreover, HCWs may feel a professional responsibility to model healthy behaviors to their patients, further motivating them to adopt healthier habits [41, 42].

In our study, 40% of the general population exhibited an intermediate to high risk of ASCVD (≥ 7.5%), surpassing the pooled percentage reported in a meta-analysis (20.8%) and closely aligning with the findings from a study conducted in Iran, where approximately 35% of the population had a risk of ≥ 7.5% [43, 44]. Other studies in the literature have used different cut points to classify ASCVD risk categories, with many considering 10% as the threshold for high risk [45]. Additionally, various cardiovascular risk calculators have been employed, which may limit our ability to directly compare our findings with those of other studies, and these comparisons should be interpreted with caution. For example, A study using the Framingham risk score (FRS) reported 14% and 26% of participants with 10–20% and ≥ 20% 10-year risk, respectively [46]. In comparison, our study found lower rates of 11.6% and 3.6% among the general public, aligning more closely with a Nigerian study reporting 8.5% and 14.6% using WHO charts [47].

Among HCWs, 1.5% had a high 10-year risk of ≥ 20% which exceeded the results reported in a Nigerian study (0.7%) and was lower than that reported in a Malaysian study (12.8%) [48, 49]. Specifically, among physicians, 27.5% were identified to have an intermediate to high 10-year risk, which was approximately double the figure reported among cardiac care physicians in Pakistan [50]. This discrepancy could be attributed to cardiac physicians being more proactive in adopting healthy lifestyles due to their professional expertise as cardiologists.

Comparing the 10-year risk between HCWs and the public, the study found that the general public group had a significantly higher risk. A study conducted in Canada yielded similar findings, revealing that practicing physicians had a lower prevalence of cardiovascular risk factors, and a reduced likelihood of major adverse cardiovascular events compared to the general population [14]. These results are indirectly supported by other studies that have compared the prevalence of cardiovascular risk factors between HCWs and the general public, rather than calculating the ASCVD risk. These studies consistently show that HCWs have healthier patterns of CVD risk factors and lifestyles [51–54]. This can be explained by several factors. HCWs are more likely to have better access to health information and preventive care, given their professional background and work environment [50]. This knowledge leads to healthier lifestyle choices, such as better diet, regular physical activity, and avoidance of smoking, all contributing to lower ASCVD risk. Additionally, HCWs often undergo regular health screenings as part of their employment, facilitating early detection and management of cardiovascular risk factors [55].

Upon assessing the predictors of having intermediate to high 10-year risk, lifestyle status was significantly associated with the outcome in both groups as participants with unhealthy lifestyles were significantly more likely to have an intermediate-high risk. This observation emphasizes the importance of lifestyle factors, such as diet, physical activity, and smoking habits, in influencing cardiovascular health outcomes among both HCWs and the general population. Our study revealed that nurses and midwives were approximately 11 times more likely than physicians to have intermediate to high 10-year risk. These professionals often work in physically demanding environments, which can lead to physical and mental fatigue. Additionally, their roles typically involve shift work, which has been associated with disrupted circadian rhythms and adverse metabolic effects [56–58]. The stress associated with patient care, coupled with potentially lower access to health information and preventive measures compared to physicians, may also contribute to their increased risk. This highlights the need for targeted interventions and support systems to mitigate cardiovascular risk among nurses and midwives, addressing both the occupational hazards and promoting healthier lifestyle practices within these groups.

Similar to the general public group, HCWs with healthier lifestyles were less likely to have intermediate to high 10-year cardiovascular disease risk. Furthermore, our analysis revealed that HCWs who lived alone were about 7 times more likely to have intermediate to high 10-year cardiovascular disease risk compared to those living with families. Individuals living alone may experience higher levels of social isolation and loneliness, which have been linked to poorer cardiovascular health outcomes [59]. Moreover, living alone may also influence lifestyle behaviors, such as dietary choices with higher chances of dining out at restaurants or ordering junk food which can impact cardiovascular risk especially that most HCWs in Qatar are expatriates who came overseas, without their families. The presence of social support and familial relationships in households where HCWs live with their families may contribute to better stress management and coping mechanisms, thereby reducing the risk of cardiovascular disease. Overall, these findings emphasize the importance of considering social determinants of health, including living arrangements, in understanding and addressing cardiovascular risk among HCWs.

Based on the study findings, policymakers are encouraged to develop and implement national and community-based strategies that promote healthier lifestyles among Qatar’s general public. Priority should be given to targeted interventions focusing on improving dietary habits and supporting smoking cessation. Additionally, enhancing health education and public awareness about cardiovascular risk factors can significantly contribute to lowering ASCVD risk. These efforts should be integrated into broader public health initiatives to ensure sustained impact and community-wide engagement.

The study provides a comprehensive assessment and valuable insights into ASCVD risk among both the general public and HCWs in Qatar. This is the first study to calculate ASCVD risk among the general population as well as among HCWs in Qatar. However, the study has several limitations. For the HCWs group, participants were exclusively sampled from HMC. Although HMC is the main healthcare provider in Qatar, it primarily offers secondary and tertiary care, which may limit the generalizability of the findings. One of the limitations of our study is the potential for social desirability bias, particularly within the HCWs group. Due to their medical knowledge and awareness of healthy lifestyle practices, HCWs may be more inclined to provide responses that they believe are expected or socially acceptable, rather than reflecting their true behaviors or opinions. This bias could lead to an overestimation of their adherence to healthy lifestyle practices and, consequently, an underestimation of their actual ASCVD risk. Reliance on self-reported data, especially for lifestyle habits, might have introduced recall biases. Additionally, the cross-sectional design of the study limited its ability to establish causal relationships or observe changes in ASCVD risk over time.

## Conclusions

This study showed that HCWs have significantly lower 10-year ASCVD risk compared to the general public. This difference is largely attributed to healthier lifestyle habits observed among HCWs, particularly in terms of dietary and smoking habits. Lifestyle habits emerged as the primary independent predictor of higher ASCVD risk in both groups. Additionally, among HCWs, living alone and working as a nurse were identified as significant independent predictors of a higher 10-year ASCVD risk. These findings provide critical insights for policymakers. They can guide the development of national and community-based strategies to promote healthy lifestyles among Qatar’s general public, focusing on diet and smoking cessation. Enhancing health education and awareness can play a vital role in reducing ASCVD risk in the general public.

## Data Availability

Data will be available upon reasonable request from the corresponding author.
